# Isoenergetic Feeding of Low Carbohydrate-High Fat Diets Does Not Increase Brown Adipose Tissue Thermogenic Capacity in Rats

**DOI:** 10.1371/journal.pone.0038997

**Published:** 2012-06-13

**Authors:** Matthias J. Betz, Maximilian Bielohuby, Brigitte Mauracher, William Abplanalp, Hans-Helge Müller, Korbinian Pieper, Juliane Ramisch, Matthias H. Tschöp, Felix Beuschlein, Martin Bidlingmaier, Marc Slawik

**Affiliations:** 1 Endocrine Research Unit, Medizinische Klinik und Poliklinik IV, Klinikum der LMU, Munich, Germany; 2 Department of Medicine, Metabolic Diseases Institute, University of Cincinnati, Cincinnati, Ohio, United States of America; 3 Institute for Medical Informatics, Biometry and Epidemiology, Ludwig-Maximilians University, Munich, Germany; 4 Clinic of Small Animal Surgery and Reproduction, Centre of Clinical Veterinary Medicine, Ludwig-Maximilians University, Munich, Germany; 5 Institute for Diabetes and Obesity, Helmholtz Centre for Health and Environment and Technical University, Munich, Germany; State University of Rio de Janeiro, Biomedical Center, Institute of Biology, Brazil

## Abstract

Low-carbohydrate, high-fat (LC-HF) diets are popular for inducing weight loss in overweighed adults. Adaptive thermogenesis increased by specific effects of macronutrients on energy expenditure has been postulated to induce this weight loss. We studied brown adipose tissue (BAT) morphology and function following exposure to different LC-HF diets.

**Methods:**

Male Wistar rats were fed a standard control diet ad libitum or pair-fed isoenergetic amounts of three experimental diets for 4 weeks. The diets had the following macronutrient composition (% metabolizable energy: carbohydrates, fat, protein): control (64.3/16.7/19), LC-HF-low protein (LC-HF-LP, 1.7/92.8/5.5), LC-HF-normal-protein (LC-HF-NP, 2.2/78.7/19.1), and a high fat diet with carbohydrates (“high fat”, 19.4/61.9/18.7).

**Results:**

Body weight gain was reduced in all pair-fed experimental groups as compared to rats fed the control diet, with more pronounced effect in rats on LC-HF diets than on the high fat diet with carbohydrates. High fat diets increased expression of PGC1α and ADRB3 in BAT indicating higher SNS outflow. However, UCP1 mRNA expression and expression of UCP1 assessed by immunohistochemistry was not different between diet groups. In accordance, analysis of mitochondrial function in-vitro by extracellular flux analyser (Seahorse Bioscience) and measurement of inducible thermogenesis in vivo (primary endpoint), explored by indirect calorimetry following norepinephrine injection, did not show significant differences between groups. Histology of BAT revealed increased lipid droplet size in rats fed the high-fat diet and both LC-HF diets.

**Conclusion:**

All experimental diets upregulated expression of genes which are indicative for increased BAT activity. However, the functional measurements in vivo revealed no increase of inducible BAT thermogenesis. This indicates that lower body weight gain with LC-HF diets and a high fat diet in a pair-feeding setting is not caused by increased adaptive thermogenesis in BAT.

## Introduction

Adherence to low carbohydrate-high fat (LC-HF) diets has been associated with higher short term weight loss [1], [2] and a more favorable lipid profile [3], than conventional low-fat diets. Although more recent studies did not detect a significantly lower body weight after long-term intake of low-carbohydrate, high-fat diets compared to those with low-fat and normal carbohydrate content [4], [5], low carbohydrate diets enjoy unaltered popularity.

Several mechanisms have been suggested to cause weight loss in LC-HF diets: the induction of ketosis and the associated loss of energy via ketone bodies excreted in urine, suppression of appetite due to circulating ketones, or increased energy expenditure due to adaptive thermogenesis [6], [7]. However, these potential mechanisms and the effectiveness of low carbohydrate diets have been questioned [8]. Moreover, we have recently shown that the loss of energy via excretion of ketone bodies is quantitatively negligible in rats fed a ketogenic diet and does not contribute substantially to overall energy expenditure [9]. In addition, ketosis was not associated with weight loss in humans [1]. While there is up to now no evidence of increased energy expenditure in humans on a ketogenic diet, increased energy expenditure was demonstrated in mice, but not rats fed a ketogenic diet with ad libitum access to food [10], [11].

Brown adipose tissue (BAT) is a metabolically highly active tissue with the unique capability to dissipate chemical energy into thermal energy (heat) needed to defend core body temperature in cold environments. While it has been known to be of major importance in rodents and newborn humans, it was only recently that several publications highlighted its relevance in adults and its possible connection to overweight and obesity [12], [13], [14].

About 30 years ago Rothwell and Stock observed that rats fed palatable foods high in fats and refined carbohydrates (so called “cafeteria diet”) in addition to the standard laboratory chow ad libitum, developed hyperphagia, but gained less weight than expected from the energy content of the ingested food. Furthermore, they discovered that the animals exhibited increased BAT weight and increased norepinephrine stimulated thermogenesis, which led to the conclusion that additional energy intake in “cafeteria diet” is partially dissipated by BAT in an attempt to counteract obesity [15].

It is an ongoing debate whether high fat diets increase inducible adaptive thermogenesis in BAT. Moreover, it is unknown whether the consumption of high amounts of calories per se or individual macronutrients, such as fat, trigger increased adaptive thermogenesis.

In order to systematically dissect the effects of the three macronutrients fat, carbohydrates and protein on BAT recruitment and function in rats, we compared the effects of pair-feeding iso-energetic amounts of four differentially composed diets. Using a rat model and a pair-feeding setting, we investigated the effects of macronutrients on body-weight gain, brown adipose tissue and adaptive thermogenesis capacity, which was the major objective of this study.

## Methods

### Animal procedures

Male Wistar rats (Wistar Unilever, Harlan-Laboratories, Borchen, Germany, 10-week old at delivery, 12-week at diet start (after acclimation), 16-week old at dissection) were housed in individual cages in a thermo-controlled environment (21.3±0.6°C, humidity 60±10%) maintained on a 12-hour light-dark cycle throughout the study. All animals received ad libitum access to water and standard laboratory chow (CH) for the first 14 days following delivery to allow acclimation to the new environment. Body weight (BW) and 24 hour food intake were measured daily (Sartorius Competence CP2201, Goettingen, Germany) to the nearest 0.1 g one hour before the onset of the dark period. After the acclimation period rats were weight matched, split into four groups and pair fed (control vs. intervention diets) for 4 weeks with diets differing significantly in macronutrient content composition. The diets (Kliba Nafag – business unit of PROVIMI KLIBA SA, Kaiseraugst, Switzerland) had the following macronutrient composition (% metabolizable energy: carbohydrates, fat, protein): control, 64.3/16.7/19.0; low-carbohydrate-high-fat-low protein (LC-HF-LP); 1.7/92.8/5.5, low carbohydrate-high-fat-normal-protein (LC-HF-NP); 2.2/78.7/19.1, normal carbohydrate-high fat-normal protein (high fat) 19.4/61.9/18.7. All of these diets used identical macro-nutrient sources (protein-source: casein; fat-source: beef tallow; carbohydrate-source: starch), therefore allowing the precise comparison of differences in macronutrients. The metabolizable energy (ME) content of all diets was calculated by the Atwater formula. This formula can be used for a precise calculation of ME as long as the digestibility of macronutrients (i.e. gastrointestinal uptake) is similar in all diets. To confirm the Atwater-formula based calculation of ME and assure similar digestibility of macronutrients in each diet, we had previously determined the ME content of each diet experimentally by digestion trials [9]. All diets were fed in a pair-feeding setting, since we explicitly wanted to exclude any effects which could potentially be attributed to differences in energy intake. For the pair-feeding procedure, all rats fed the control diet had ad libitum access to food and the caloric intake of the control fed animals was measured daily. Subsequently, animals fed with the experimental diets were daily given the respective amount of food to equal the amount of calories ingested by the control group. Importantly, rats fed the experimental diets consumed all of the food allocated to them.

All procedures were in accordance with the German animal welfare act and approved by the Upper Bavarian Government's ethical committee for animal experiments. Ethics committee: Upper Bavarian government, Chief Ethics committee: Dr. med. vet. B. Wirrer, Veterinärdirektorin, Sachgebiet-54, 80543 München.

### Dissection of animals

After four weeks of feeding the respective diet, rats had access to food and water for one hour at the beginning of the dark phase (lights off); then animals were fasted for 6 hours before decapitation under isoflurane anesthesia. Trunk blood was collected for further analysis and serum was stored at −80°C until analysis. Interscapular BAT samples were collected and weighed, immediately frozen on dry ice or stored in 4% paraformaldehyde (for histology). Samples used for RNA extraction were stored at −80°C until the RNA was extracted. For isolation of muscle mitochondria, samples were excised from the quadriceps femoris muscle.

### Body core temperature

Body core temperature was obtained using a Thermalert TH-5 thermometer fitted with a rectal probe, specifically developed for temperature measurement in rats (Physitemp, Clifton, NJ). Temperature of each animal was measured in the morning and in the evening. In order to minimize effects of stress and movement on core body temperature, we measured temperature early in the morning (before lights on) and late in the evening (after lights out). The individually housed rats were carefully transported in their cages to an examination table which was located in the opposite corner of the housing room. Body core temperature was then measured by experienced researchers. A reading was obtained within 20 seconds.

### Quantitative RT-PCR

1 µg of total RNA was transcribed in one run applying the SuperScript III First-Strand Synthesis SuperMix for qRT-PCR (Invitrogen, Karlsruhe, Germany).

Quantification of mRNA abundance was performed by real-time PCR detection using a Stratagene Mx3000 instrument (Stratagene, La Jolla, CA, USA) and SYBR-green as a double-stranded DNA-specific fluorescent dye (iQ SYBR Green Supermix, BioRad, Munich, Germany). Amplification primers (Table 1) were designed using NCBI's Primer BLAST software (http://www.ncbi.nih.gov/primer-blast). The expression of the respective gene of interest was normalized to 18s RNA.

**Table 1:** Primer sequences (5′-3′).

	Forward primer	Reverse primer
ADRB3	AGCTAGCCCTGTTGCGTCCA	GGAGAGTTGCGGTTCCTGGG
PGC-1α	CGATCACCATATTCCAGGTCAAG	CGATGTGTGCGGTGTCTGTAGT
PPARγ1	CCCAGAGCATGGTGCCTTCGC	TCCGAAGTTGGTGGGCCAGA
PPARγ2	AGGCTGCAGCGCTAAATTCATCT	TCCGAAGTTGGTGGGCCAGA
PRDM-16	AGGTGAGGTCTGCCACAAGT	CCCGGCGTGTAATGGTTCTT
UCP1	CGAGCCAAGATGGTGAGTTCGACA	GTGGTGATGGTCCCTAAGACACCT
18S RNA	GGGAGGTAGTGACGAAAAATAACAAT	TTGCCCTCCAATGGATCCT

### HE-staining and Immunohistochemistry (IHC)

For morphological evaluation, tissues were dehydrated, embedded in paraffin, sectioned, and stained with hematoxylin and eosin (H&E) following standard procedures.

For UCP1 immunohistochemistry, paraffin-embedded sections were rehydrated, blocked with 0.3% H_2_O_2_ in methanol for 10 min, and incubated with blocking buffer for 15 min. UCP1 was immunolocalized overnight at 4°C by means of a rabbit polyclonal antibody (1∶500, Sigma U6382), in a dilution of 1∶500 in blocking buffer containing 3% BSA (Roche), 5% goat serum (Jackson ImmunoResearch Laboratories), and 0.5% Tween 20. After rinsing for 15 min in PBS, secondary antibody (goat anti-rabbit biotinylated IgG (Vector Laboratories, Burlingame, CA, USA)) was applied for 30 min at room temperature. For the visualization of the bound antibody, Vectastain Elite ABC system (Vector Laboratories) and Sigma Fast diaminobenzidine (Sigma) were used.

### Morphometry

Lipid droplet size was assessed with ImageJ Version 1.43 (National Institutes of Health, Bethesda, MD). Images were obtained at a resolution of 10 megapixels, converted to 8-bit grayscale and the grayscale image was inverted, so that lipid droplets were dark gray. The image was then converted to binary with the threshold set to the middle of the grayscale range (127/255). A watershedding algorithm was executed and particles counted automatically using the built in particle counting method of ImageJ. Minimal particle size was set to 1000 square pixels and circularity to 0.3 to 1.0, with circularity being defined as 4π(area/perimeter^2^).

### Isolation of Mitochondria

BAT and M. quadriceps samples were excised and transferred to a 2 ml microcentrifuge tube with 1 ml Isolation buffer 1 (IB1), containing 210 mM mannitol, 70 mM Sucrose, 5 mM HEPES, 1 mM EGTA, 0.5% fatty acid free BSA. Tissue was grinded, centrifuged at 700×g for 10 minutes. The supernatant was then transferred to a fresh tube and centrifuged at 14000×g for 10 min. The obtained pellet was resuspended in IB1 and centrifuged again at 10000×g for 10 min. The same procedure was repeated with Isolation Buffer 2 (IB2), containing 210 mM mannitol, 70 mM sucrose, 10 mM MgCl_2_, 5 mM potassium diphosphate, 10 mM MOPS, and 1 mM EGTA. The final mitochondrial pellet was then resuspended in 50–200 µl IB2.

### Measurement of mitochondrial respiration by Seahorse technique

An XF 24 extracellular flux analyser was used to determine mitochondrial function (XF 24, Seahorse Bioscience, http://www.seahorsebio.com/company/about.php). The validity of this method in comparison to classical Clark electrode oxygraph measurements has been proven recently [16]. Before measurement, calibration for 10 min (37 C) was performed. Mitochondria from BAT and muscle were isolated and 15 µg per well transferred in 500 µl MAS-1 buffer (Sucrose 70 mM, Mannitol 220 mM, KH2PO4 5 mM, MgCl2 5 mM, KCl 50 mM HEPES 2 mM, EDTA 1 mM, FA Free BSA 0.1% (add fresh to vol. used), pH 7.4). In order to analyze respirometry the following substances were added: State II: Pyruvate 5 mM, Maleate 5 mM; State III: ADP 0.25 mM; State IVo: Oligomycin 1 µM; FCCP (complete uncoupling), 300 nM.

### Norepinephrine stimulated thermogenesis

After 21 days feeding of the respective diet, norepinephrine stimulated thermogenesis was analyzed during the afternoon following 6 h fasting, preceded by access to fodder for two hours in order to be prepared until recovering from anesthesia. We deliberately chose these feeding/fasting conditions in order to ensure equal gastrointestinal filling for all animals and to avoid confounding the energy expenditure measurements by acute thermogenic effects of feeding (i.e. energy expenditure necessary to digest and absorb nutrients). Rats were sedated with pentobarbital (45 mg/kg; i.p.) and O_2_ consumption and CO_2_ production were analyzed every 9 minutes via an indirect calorimetry system (CaloSys, TSE Systems GmbH, Bad Homburg, Germany) in order to determine energy expenditure (EE) and respiratory quotient (RQ). Data on EE were normalized to the respective metabolic mass (body weight^0.75^) of individual rats. Basal energy expenditure was analyzed for 27 min, before norepinephrine (1 mg/kg body weight) was injected intra peritoneally. Subsequently, oxygen consumption and carbon dioxide production (CO_2_) was analyzed for another 60 min.

### Statistics

Seven animals per group (n = 28) were investigated in the experiments. If not otherwise stated, the analyses deal with the complete group numbers.

The endpoint regarding the primary objective was defined as the averaged maximum energy expenditure measured at 43, 52, and 61 minutes after injection of norepinephrine. It was analyzed setting up a closed testing procedure to adjust the type I error level of α = 5% for multiple comparisons of groups. In doing so, Dunnett's test comparing the individual diet groups to control was enhanced to all group comparisons. For the purpose of sensitivity analyses, closed testing based on one-way ANOVAs was performed in addition.

Sample size calculation aimed at significance (multiple comparison of groups with strong control of type I error at two-sided level of α = 5%) to detect an effect size of 2.4 kcal/h/kg^0.75^ with a power of 80% assuming a conservative standard deviation of 1.6 kcal/h/kg^0.75^). Originally, the experiment was planned with 1 control and 6 diet intervention groups, resulting in a group size of 11 animals per group. For technical reasons the number of intervention groups was reduced to 3, allowing for a slight reduction of animals per group. With the feasible number of 7 rats per group at least a power of 60% was expected.

The effect of diet on body weight (repeated measurements growth curve) was analyzed by two-way ANOVA and delta weight-gain with the same procedure as the primary objective (closed testing based on Dunnett tests and one-way ANOVAs).

For RQ analysis, RQ values were calculated as the average of three time points at baseline (before injection of NE) and as the average of three time points during the plateau phase of maximum BAT activity (43, 52, and 61 minutes after injection of NE).

RQ values, gene expression levels, body core temperature, and fat pad weights were analyzed with the same closed testing procedures (enhanced Dunnett testing and ANOVAs). Lipid droplet size was analyzed with the same closed testing strategies. However, as data did not follow a normal distribution, the Kruskal-Wallis-test was used instead of one-way ANOVA.

If not otherwise stated all data are depicted as means ± SEM. * p<0.05, ** p<0.01, *** p<0.001 (see results section). Calculations were performed with GraphPad Prism Version 5.04 (GraphPad, La Jolla, CA) and SPSS Version 20 (IBM, Armonk, NY).

## Results

### Low-carbohydrate, high-fat diets slow body weight gain

**Figure 1:**
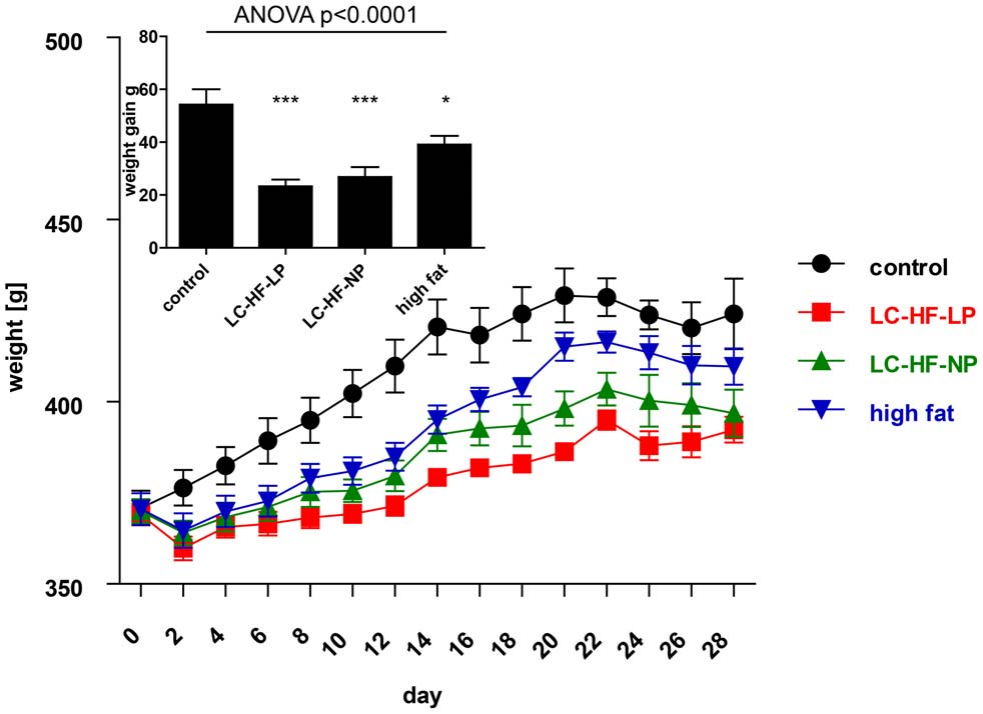
Weight curves of rats on the control diet (circles, fed ad libitum), compared to the three experimental diets (iso-energetic pair feeding): low-carbohydrate high-fat, low-protein diet (LC-HF-LP; squares), low-carbohydrate high-fat, normal-protein diet (LC-HF-NP; upward triangles) or high fat diet (downward triangles); p<0.0005, repeated measurements ANOVA. The cumulative average weight gain for each group during the four week feeding period is given in the insert, analyzed by global one-way ANOVA and Dunnett tests for pairwise comparison vs. control: * p<0.05, *** p<0.001. Data are shown as means±SEM; n = 7 animals/group.

All animals gained weight during the 4 weeks of ad libitum (control) or iso-energetic feeding (intervention; Fig. 1). Diet had a significant effect on body weight gain (global p<0.0005, repeated measurements ANOVA.). Accordingly, the mean weight gain after four weeks was significantly different among the groups (one-way ANOVA, p<0.0001). Body weight gain was significantly lower in all rats fed the experimental diets as compared to the control group (control diet: 54.2±5.8 g; LC-HF-LP, 23.3±2.5 g, Dunnett p<0.001; LC-HF-NP, 26.8±3.7 g, p<0.001; high fat diet, 39.1±3.3 g, p = 0.026; Fig. 1, insert).

### LC-HF-LP diet fed animals have higher interscapular brown adipose tissue weight

**Figure 2:**
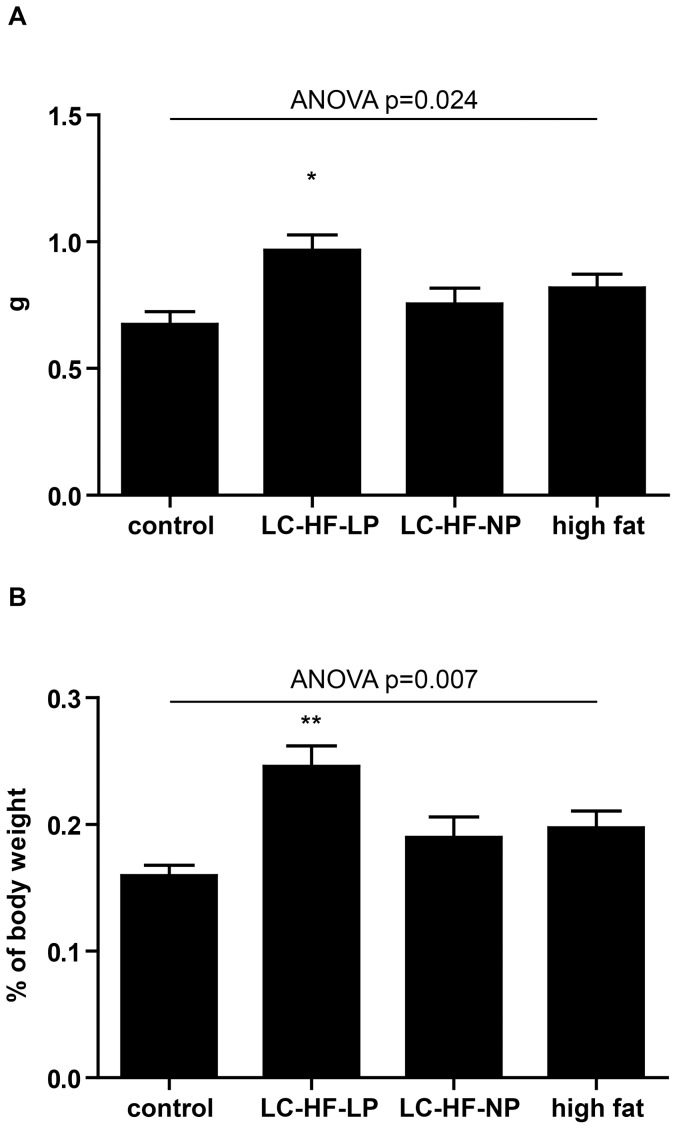
Weight of interscapular brown adipose tissue (iBAT) in rats fed control or experimental diets (LC-HF-LP, LC-HF-NP, high fat). A: total weight (absolute); B: percentage of body weight (relative). Data are shown as means±SEM, n = 7/group; analyzed by global one-way ANOVA and Dunnett tests for pairwise comparison vs. control. * p<0.05, ** p<0.01 vs. control.

Recently, we had examined the effects of LC-HF diets on body composition using NMR and found an increased total body fat content using these experimental diets [17]. This prompted us to weigh the interscapular brown adipose tissue (iBAT) pad, which is the biggest and most discernible BAT depot in rodents. Diets had a significant effect on absolute and relative iBAT weight, ANOVA p = 0.024 and p = 0.007, respectively. Direct comparison between groups revealed that only rats fed the LC-HF-LP diet had significantly higher iBAT weight (Fig. 2 A, B), both in absolute (control 0.67±0.05 g, LC-HF-LP 0.97±0.06 g, p = 0.011) as well as in relative terms when normalized to body weight (control 0.16±0.008%, LC-HF-LP 0.25±0.016%, p = 0.003). The absolute and relative weights of iBAT in animals fed LC-HF-NP and high fat diets were not significantly different when compared to the control group (LC-HF-NP 0.75±0.06 g, p = 0.742; 0.19±0.015%, p = 0.462, high fat diet 0.82±0.05 g, p = 0.226; 0.2±0.01%, p = 0.193.; Fig. 2B).

### Body core temperature

**Figure 3:**
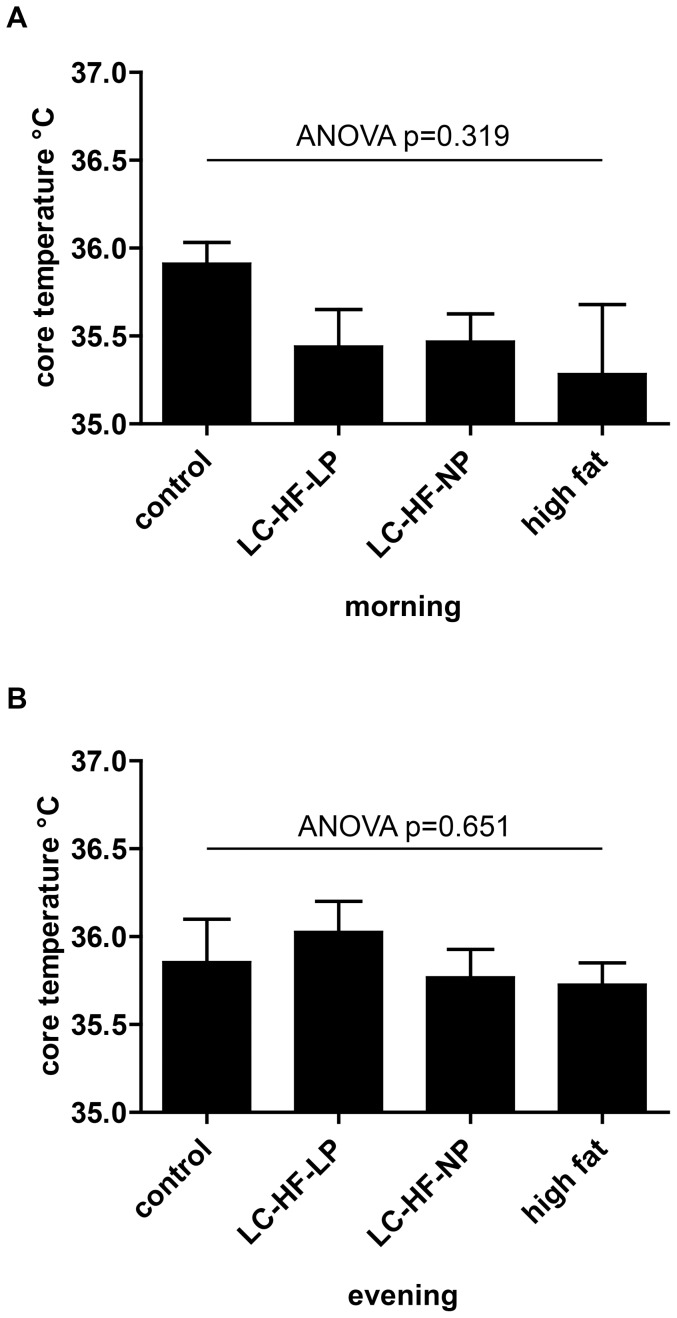
Body core temperature in rats fed control or experimental diets (LC-HF-LP, LC-HF-NP, high fat). A: morning, B: evening. Data are shown as means±SEM; n = 7/group; analyzed by global one-way ANOVA and by Dunnett tests for pairwise comparison vs. control.

As changes in BAT mass might influence body core temperature, temperature measurements were performed during morning and evening. Results are shown in figure 3. Registered body core temperature in the morning was 35.9±0.1°C in control, 35.4±0.2°C in LC-HF-LP (p = 0.395 vs. control), 35.5±0.2°C in LC-HF-NP (p = 0.443), and 35.3±0.4°C in high fat (p = 0.189) diet fed animals, respectively, the differences not being statistically significant (ANOVA p = 0.319). Similarly, the evening temperatures were not significantly different between groups: control 35.9±0.2°C, LC-HF-LP 36.0±0.2°C (p = 0.837), LC-HF-NP 35.8±0.2°C (p = 0.974), high fat 35.7±0.1°C (p = 0.920), ANOVA p = 0.651.

### Histology of brown adipose tissue

**Figure 4:**
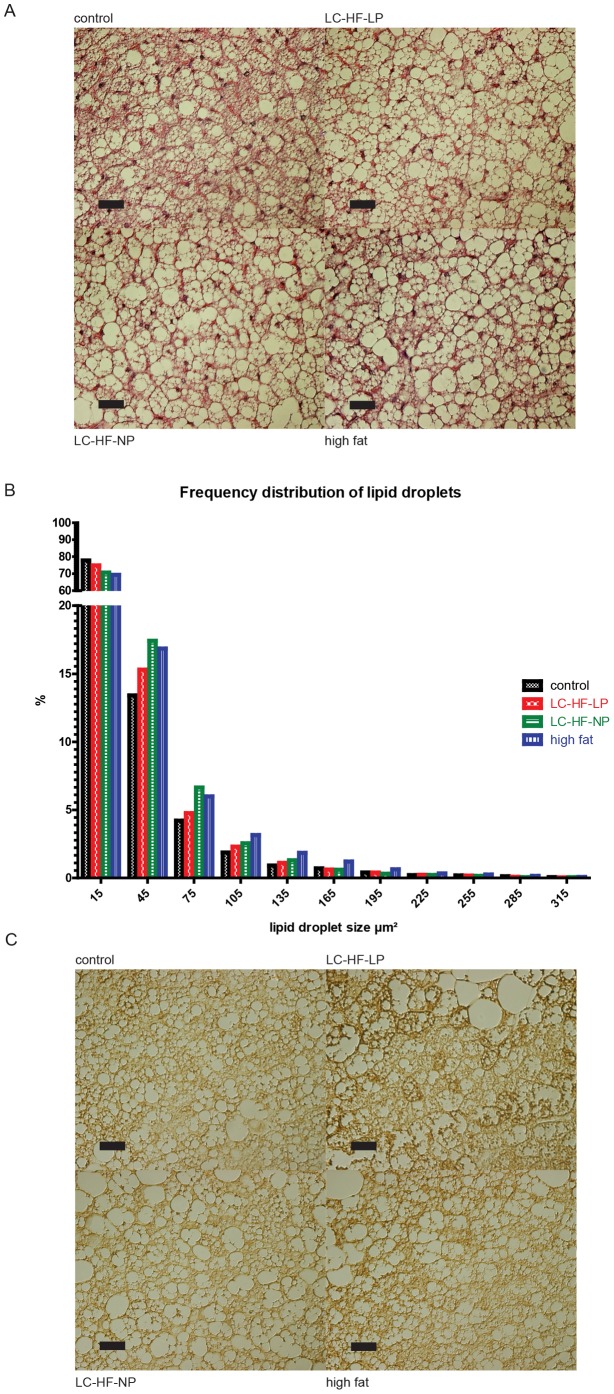
Histology of iBAT in rats fed control or experimental diets (LC-HF-LP, LC-HF-NP, high fat). A: H & E staining. B: Morphometric analysis of lipid droplet size distribution. p<0.0001 for each experimental diet vs. control, analysed with Kruskal-Wallis test. C: immunohistochemistry with UCP1 antibody. Expression of UCP1 appears in a brownish colour. Black bar in photomicrographs represents 30 µm scale.

In order to comprehend specific effects of macronutrient composition on BAT we analyzed the tissue histologically: Hematoxylin and eosin (H&E) staining revealed differences in BAT morphology (Fig. 4A), showing a higher proportion of larger lipid droplets in the experimental diets. This was confirmed using morphometric analysis assessing the size of fat droplets in a high power field and subsequent statistical analysis (Kruskal-Wallis p<0.001 for all group comparisons and direct comparisons of the experimental diet vs. control; Fig. 4B). In addition, immunohistochemistry of UCP1 expression revealed no obvious differences of this thermogenic marker protein between the different diet groups (Fig. 4C). To corroborate the findings of increased lipid droplet size in the experimental groups we determined amount of lipid with an imaging based method of the interscapular BAT (details in Methods S1). The surface covered by lipids per high power field was significantly lower in the control (62.9±2.2%) than in the experimental groups (LC-HF-LP 69.8±0.9%, LC-HF-NP 69.9±0.9%, and high fat diet 67.4±1.9%), ANOVA p = 0.037 (Table S1).

### High-fat feeding induces changes in the expression of genes regulating BAT development and adaptive thermogenesis

**Figure 5:**
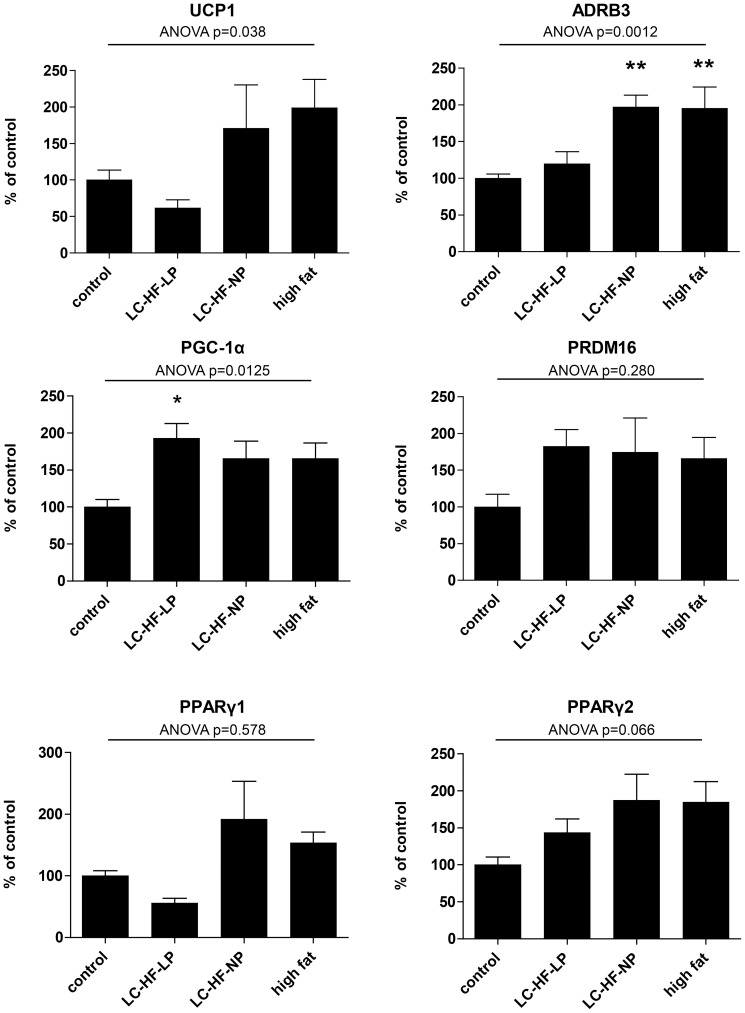
Quantitative real time PCR in iBAT following control or experimental diets (LC-HF-LP, LC-HF-NP, high fat). ADRB3: β3 adrenergic receptor. Gene expression was normalized to 18 S rRNA. Data are shown as means±SEM; n = 7/group; analyzed by global one-way ANOVA and by Dunnett tests for pairwise comparison vs. control. * p<0.05, ** p<0.01 vs. control.

BAT thermogenesis is dependent on the induction of specific genes responsible for driving BAT development, transmission of sympathetic nervous system (SNS) signalling and uncoupling in mitochondria. Speculating that changes in macronutrient composition might induce adaptive thermogenesis in BAT, we measured the expression of UCP1 mRNA in brown adipose tissue samples. While the global ANOVA indicates a significant influence of diet on UCP1 expression (p = 0.038), the differences failed to reach significance for the comparisons of control vs. experimental diets (Fig. 5).

The expression of the β-3-adrenoreceptor was significantly influenced by the different diets, ANOVA p = 0.0012. In comparison to control (100±15%), ADRB3 expression was not significantly affected in LC-HF-LP fed animals (119±17%, p = 0.791 vs. control). In contrast ADRB3 levels were markedly increased in LC-HF-NP (197±16%, p = 0.004) and high fat diet (195±16%, p = 0.004) fed animal, suggesting a central nervous system regulated increased SNS activity.

To gain further insight into the possible effects of the experimental diets on BAT we measured the expression of PPARγ coactivator-1α (PGC-1α), a master regulator of mitochondrial biogenesis and oxidative metabolism, as well as PRDM16 and PPARγ isoforms 1 and 2, transcription factors essential to the development of BAT. PGC-1α expression was increased in all animals fed with the experimental high fat diets (control 100±10%; LC-HF-LP 193±20%, p = 0.004 vs. control; LC-HF-NP 165±23%, p = 0.072; high fat 166±21%, p = 0.058; ANOVA p = 0.0125).

Expression of PRDM16, a transcription factor determining brown adipocyte differentiation was not significantly different between the control and experimental groups. As compared to the control group (100±8%) PPARγ1 expression was not affected by the experimental diets: LC-HF-LP group 56±8%, p = 0.683 vs. control; LC-HF-NP 192±61%, p = 0.161; high fat diet 153±18%, p = 0.578. ANOVA indicated significantly different means (p = 0.039), but none of the groups was significantly different from control. The expression of PPARγ isoform 2 was not significantly affected by the experimental diets either (ANOVA p = 0.066).

### Respiratory function of mitochondria is not altered after four weeks of high-fat feeding

**Figure 6:**
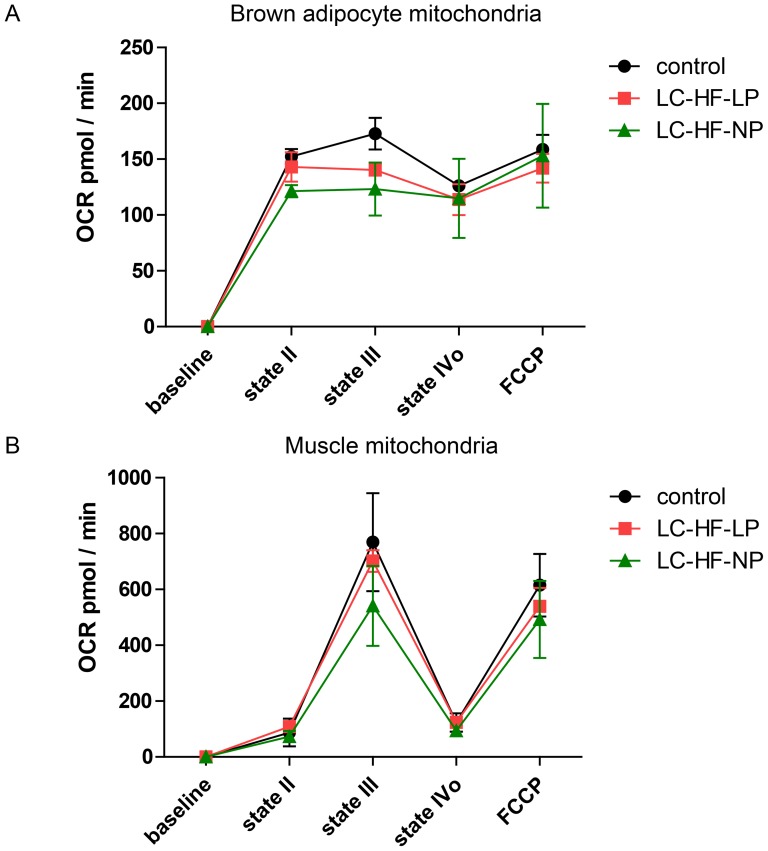
Respirometry. Measurement of mitochondrial oxygen consumption using microplate based extracellular flux analyzer in tissues from rats fed the control or experimental diets (LC-HF-LP, LC-HF-NP). A: brown adipocyte mitochondria. B: muscle mitochondria. Stage II, basal; stage III, ADP; stage IVo, oligomycin. N = 3/group.

To further explore the mitochondrial function of brown adipocytes in-vitro we used a microplate based extracellular flux analyzer (Seahorse XF 24). As shown in figure 6, mitochondrial function of BAT and muscle did not differ significantly between diets explored. Addition of ADP (Stage III) increased oxygen consumption from stage II in muscle but not in brown adipocytes mitochondria (BAM). Oligomycin nearly completely blocked respiration in muscle, but did not result in changes in BAM (stage IVo). FCCP again increased OCR in muscle by total uncoupling, but did not increase oxygen consumption in BAM further.

### Isoenergetic high-fat feeding does not increase inducible thermogenesis

Basal and norepinephrine (NE) induced energy expenditure in animals was measured using indirect calorimetry, the latter reflecting maximal thermogenic activity in BAT. Under baseline conditions the respiratory quotient (RQ) of the animals fed LC-HF-LP (0.670±0.014, p<0.008 vs. control) and LC-HF-NP (0.665±0.006, p = 0.01) diets was significantly lower than the RQ of the control group (0.741±0.018). The RQ of rats fed high fat diet was not significantly different from control (0.714±0.016, p = 0.466). The global 4 group ANOVA resulted in p = 0.0061. Following NE injection RQ increased in all experimental and the control groups. The RQ of LC-HF-LP (0.769±0.010, p<0.001), LC-HF-NP (0.769±0.009, p<0.001) and high-fat diet fed animals (0.800±0.005, p = 0.001) were significantly lower than the control group (0.857±0.0141, ANOVA p<0.001) proving metabolism of lipids as the main substrate in BAT following activation.

**Figure 7:**
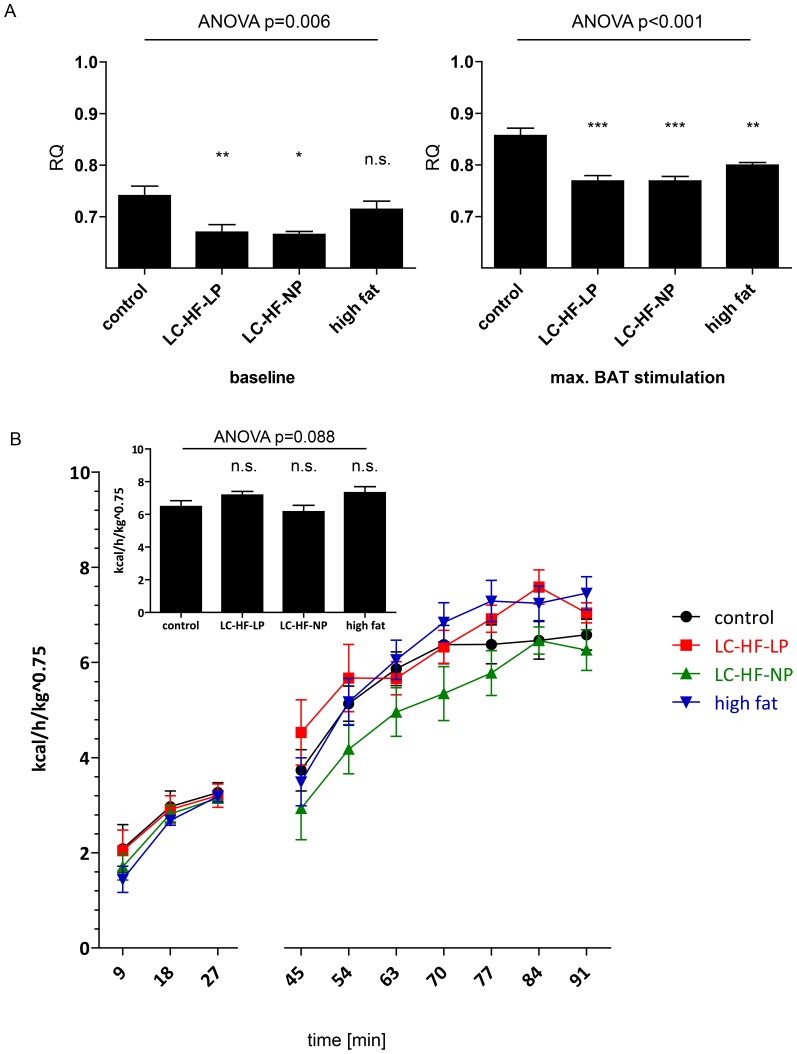
In-vivo measurement of maximal adaptive thermogenic capacity in rats fed the control or experimental diets (LC-HF-LP, LC-HF-NP, high fat). A: respiratory quotient after feeding control or experimental diets for 4 weeks: basal and following norepinephrine injection. B: Basal and norepinephrine stimulated energy expenditure (EE), expressed as kcal/h/kg BW^0.75^. The time points 0 min and 36 min are not given, as cages had to be opened for animal handling and measures of RQ and EE are not reliable at these time points. The insert shows the mean EE of timepoints 77, 84 and 91 min. Data are shown as means±SEM; n = 7 (control), 6 (LC-HF-LP), 5 (LC-HF-NP), 7 (high fat). Statistical analysis was performed on the average of three time points at baseline and during the plateau phase after NE injection, respectively, using global one-way ANOVA and Dunnett tests for pairwise comparison vs. control.

Energy expenditure during the time course of the experiment is shown in figure 7B. Following the injection of norepinephrine, total energy expenditure doubled within approximately 40 min in all groups. During the experiment one animal with LC-HF-LP diet showed implausibly high EE (more than six times SD above the other animals) and two animals in the LC-HF-NP group had very low EE values indicating anesthesia overdose; these values were excluded from the final data analysis. In contrast to our initial hypothesis no significant differences in maximal inducible thermogenesis between control and experimental diet groups could be detected (insert Fig. 7B). Maximum energy expenditure after injection of norepinephrine was as follows: control 6.48±0.36 kcal/kg^0.75^/h, LC-HF-LP 7.18±0.22 kcal/kg^0.75^/h, LC-HF-LP vs. control p = 0.202, LC-HF-NP 6.12±0.39 kcal/kg^0.75^/h, LC-HF-NP vs. control p = 0.987, high fat diet 7.33±0.37 kcal/kg^0.75^/h, high fat diet vs. control  = 0.703 (LC-HF-LP: n = 6; LC-HF-NP: n = 5; see Discussion), the global 4 group ANOVA resulted in p = 0.088.

## Discussion

Feeding a high carbohydrate, high fat diet ad libitum has been considered to activate and recruit BAT and thereby reduce metabolic efficiency [15], [18], [19]. We have previously reported that LC-HF diets effectively induce ketosis and increase serum levels of FGF-21 and leptin and decrease insulin levels in rats [17], [20], [21]. Based on other reports linking FGF-21 [22], [23] and leptin to BAT activity [24] we had hypothesized that LC-HF diets could potentially increase BAT thermogenic capacity.

In our study pair feeding of low-carbohydrate/high fat diets (LC-HF-LP or LC-HF-NP) as well as a high-fat diet with intermediate amounts of carbohydrates (high fat) compared to a control diet rich in carbohydrates and low in fat resulted in significantly lower body weight gain. This effect was most pronounced in the LC-HF-LP diet. The experimental diets led to an initial decrease in body weight during the first four days of the experimental phase as opposed to the control group which continuously gained weight. This initial dip in the weight curve has been described previously and is due to the consumption of muscle and liver glycogen and loss of associated water [25].

Interestingly, rats fed LC-HF-LP diet, but not other experimental diets exhibited increased interscapular BAT fat pad weights in comparison to rats on control diet, both in absolute terms and normalized to body weight. In their seminal study Rothwell and Stock had observed increased BAT weight in rats fed an energy rich, HF diet [15] and this has been reproduced by others [26], [27], [28]. However, other studies noticed no effect of high fat diets on BAT weight in rats [29], [30], [31]. In this respect our finding of subtly elevated BAT weight in the LC-HF-LP diet group only, but not in all experimental groups is in accordance with the literature. Histological analysis of BAT revealed significantly larger lipid droplets in all experimental diet groups, when compared to rats fed the control diet, which suggests rather reduced BAT activity [32].

High-fat diets can also lead to an increased number of brown adipocytes in white adipose tissue depots [33], which we did not assess systematically. Realtime PCR analysis of UCP1 expression in epididymal and perirenal white adipose tissue compartments, however, did not reveal significant differences between the control and experimental groups (data not shown). Additionally, a functionally important increase in the number of interspersed brown adipocytes would have been detected in the norepinephrine stimulation experiment.

Body core temperature can be influenced substantially by BAT activity [34] and even small variations are a good indicator of changes in brown adipose tissue derived adaptive thermogenesis. Core temperature is also subject to periodic circadian variations of up to 2°C and is influenced by food intake [35], [36]. It is therefore important to measure the temperature at clearly defined time points and under conditions minimizing stress for the animals (see methods section). Both evening and morning body core temperatures in this study did not differ significantly between groups. As shown recently, animals fed LC-HF diets have subtly lower total energy expenditure in long term measurements, probably due to reduced locomotor activity [17]. This might be reflected by the slightly lower morning temperature readings observed here in fat enriched diets.

Two recent studies demonstrated higher total energy expenditure in mice fed a ketogenic diet ad libitum [10], [11]. Kennedy and colleagues assessed BAT thermogenesis indirectly by measurement of UCP1 levels in BAT and found these to be increased in animals fed the ketogenic diet. We assessed the expression of UCP1, the hallmark of BAT, by realtime quantitative PCR and could not detect statistically significant increases of UCP1 in the experimental groups. Additionally, UCP1 immunohistochemistry (Fig. 4C) did not show obvious differences in distribution and content.

Our findings are thus in discordance with the studies cited above, but specific differences in the experimental design may have led to the disparate outcomes. First we used a uniform source of macronutrients in both control and experimental diets, which ensured that observed effects were not due to different proportions of fatty acids or amino acids. Second, and probably more important, we used iso-energetic pair feeding to ensure equal caloric intake between the control and experimental groups enabling us to analyse the influence of high fat diet in absence of overeating.

The original experimental setting of Rothwell and Stock used various palatable food items added to the standard laboratory stock diet (so called “cafeteria diet”) to induce hyperphagia. It was associated with increased BAT mass, UCP1 expression, norepinephrine turnover and oxygen consumption [15]. This classical cafeteria diet was thus not very well defined in terms of macro- and micronutrient composition. Subsequently, numerous studies were performed to further define factors leading to diet induced thermogenesis in BAT. Indeed, especially diets rich in polyunsaturated fatty acids increased UCP1 expression and energy expenditure [31], [37]. However, as evaluated in a recent systematic review by Fromme et al., neither the percentage of fat in a diet nor the duration of high fat feeding could be connected to increased UCP1 expression in BAT. Moreover, while 42 of the 62 studies analyzed reported an increase in UCP1 expression, eleven studies observed no change and nine even a decrease of UCP1 expression after high fat diet [38]. Furthermore, several studies identified an inverse relationship between protein content of the diet and BAT activity [39], [40], [41].

Adaptive thermogenesis is mediated via beta-adrenergic receptors [42]. The expression of the adrenoreceptor β3 (ADRB3) was significantly increased in rats fed the LC-HF-NP and high fat diet indicating increased SNS activity, which could activate BAT recruitment and function [43]. Rothwell and Stock established the “protein dilution hypothesis” [44], stating that animals had to overeat diets high in fat but low in protein in order to satisfy their protein requirements and would dissipate some of the excess energy through BAT thermogenesis, thereby limiting weight gain. Increased caloric intake of fat or carbohydrates is known to increase SNS activity and can thus potentially augment BAT thermogenesis [45]. Importantly, overeating was not possible for the experimental groups in our study, suggesting that rather excess energy and not high fat diet per se was responsible for the increased BAT thermogenesis reported in their studies.

Adrenergic signaling relayed by the ADRB3 induces the expression of PGC-1α, a master regulator of mitochondrial biogenesis which enhances the expression of UCP1 and other thermogenic genes [46]. Expression of this transcription factor was significantly increased in the LC-HF-LP group and tended to be increased in the two other experimental groups. The magnitude of this increase was, however, less than two-fold as compared to an approximately 10-fold increase in rodent iBAT in response to cold exposure. Interestingly, others have found a modest increase of PGC-1α expression in response to high fat diet in retroperitoneal white adipose tissue [47]. PRDM16 has recently been identified as an important transcription factor controlling the development of brown adipocytes by binding and activating PCG-1α [48]. In our experimental setting it was not significantly affected by experimental diets.

In order to assess adaptive thermogenic capacity in vivo, energy expenditure of anesthetized rats was measured under basal conditions and maximal stimulation by injection of norepinephrine, which was the primary objective of this study. This approach was established by Rothwell and Stock in their seminal study [15] and is currently considered the gold standard to explore BAT function [7], [43].

We chose to sedate the animals with pentobarbital during the assessment of NE stimulated BAT thermogenesis. In contrast to volatile anesthetics, which are known to inhibit BAT thermogenesis [49], and ketamine, which increases SNS activity [50], this injectable narcotic has been shown to have hardly any effects on SNS and BAT function [51]. The primary endpoint, mean energy expenditure (EE) during the plateau phase after the injection of norepinephrine, did not differ between control and experimental diet groups. Our study is adequately powered to detect a difference of 50% to the control group, so well below doubling of EE observed by Rothwell and Stock. The originally planned sample size (see methods) would theoretically have enabled detection of even smaller differences. However, the observed differences versus control of less than 1 kcal/h/kg^0.75^ are not worthwhile to be powered and an attempt to detect such a small difference with statistical certainty would result in an unreasonably high sample size. In addition, in vitro functional analysis of mitochondria did not reveal differences in respiratory chain activity neither in BAT nor in muscle tissue.

A limitation of our experimental setting is the fact that animals were housed at 21°C and thus not at thermoneutral conditions for rodents. BAT activity is increased at this ambient temperature in order to defend body core temperature and thus subtle changes in adaptive thermogenesis potentially caused by macronutrients might not become evident. Previously, effects of UCP1 knockout on body weight in mice became evident only after animals were housed at thermoneutrality, which is around 30°C for mice [19]. However, this notion seems rather implausible given the results of the histological analysis of BAT and the result of the NE stimulated thermogenesis. Additionally, Ma et al. performed a direct determination BAT oxygen consumption in rats fed either chow or cafeteria diet both in animals housed at thermoneutrality and at 24°C. While cafeteria diet fed rats exhibited increased total oxygen consumption, no differences in the oxygen consumption of BAT could be detected [52], [53].

Specific strengths of our study are the iso-energetic pair feeding and identical macronutrient sources for both the control and the experimental diets, enabling us to neatly assess the effects of low carbohydrate and high fat content of the diet on BAT thermogenesis. While high fat diets might have the potential to influence the expression of several genes relevant for BAT recruitment, they did not lead to functional changes of BAT activity in our experimental setting. We have summarized the different effects on weight, morphology, gene expression and function (Table S2).

In conclusion, LC-HF diets did neither increase UCP1 expression, mitochondrial function in vitro, nor BAT thermogenic capacity in vivo in this pair-feeding experiment in rats. The data presented by us provide thus evidence against a specific effect of LC-HF diets on BAT thermogenesis. Taking into account findings from previous studies in this animal model, the reduced body weight gain in rats on low-carbohydrate, high-fat diets cannot be attributed to increased energy expenditure, but is rather due to increased percentage of stored body fat and thus higher energy density in these animals [17], [20]. They are thus lighter but fatter, which should be regarded as an undesirable outcome in weight reducing diets.

## Supporting Information

Table S1 **Average amount of lipid in tissue section (percentage of high power field).**
(DOCX)

Table S2 **Major effects of the experimental diets compared to control.**
(DOCX)

Methods S1 **Quantification of surface covered by lipids.**
(DOCX)

## References

[1] Foster GD, Wyatt HR, Hill JO, McGuckin BG, Brill C (2003). A randomized trial of a low-carbohydrate diet for obesity.. N Engl J Med.

[2] Brehm BJ, Seeley RJ, Daniels SR, D'Alessio DA (2003). A randomized trial comparing a very low carbohydrate diet and a calorie-restricted low fat diet on body weight and cardiovascular risk factors in healthy women.. J Clin Endocrinol Metab.

[3] Westman EC, Yancy WS, Olsen MK, Dudley T, Guyton JR (2006). Effect of a low-carbohydrate, ketogenic diet program compared to a low-fat diet on fasting lipoprotein subclasses.. Int J Cardiol.

[4] Dansinger ML, Gleason JA, Griffith JL, Selker HP, Schaefer EJ (2005). Comparison of the Atkins, Ornish, Weight Watchers, and Zone diets for weight loss and heart disease risk reduction: a randomized trial.. JAMA.

[5] Sacks FM, Bray GA, Carey VJ, Smith SR, Ryan DH (2009). Comparison of weight-loss diets with different compositions of fat, protein, and carbohydrates.. N Engl J Med.

[6] Atkins R (1992). Dr.. Atkins' New Diet Revolution: Avon Books, New York.

[7] Cannon B, Nedergaard J (2004). Brown adipose tissue: function and physiological significance.. Physiol Rev.

[8] Astrup A, Meinert Larsen T, Harper A (2004). Atkins and other low-carbohydrate diets: hoax or an effective tool for weight loss?. Lancet.

[9] Bielohuby M, Bodendorf K, Brandstetter H, Bidlingmaier M, Kienzle E (2010). Predicting metabolisable energy in commercial rat diets: physiological fuel values may be misleading.. The British journal of nutrition.

[10] Jornayvaz FR, Jurczak MJ, Lee HY, Birkenfeld AL, Frederick DW (2010). A high-fat, ketogenic diet causes hepatic insulin resistance in mice, despite increasing energy expenditure and preventing weight gain.. American journal of physiology Endocrinology and metabolism.

[11] Kennedy AR, Pissios P, Otu H, Roberson R, Xue B (2007). A high-fat, ketogenic diet induces a unique metabolic state in mice.. Am J Physiol Endocrinol Metab.

[12] Cypess AM, Lehman S, Williams G, Tal I, Rodman D (2009). Identification and importance of brown adipose tissue in adult humans.. N Engl J Med.

[13] van Marken Lichtenbelt WD, Vanhommerig JW, Smulders NM, Drossaerts JM, Kemerink GJ (2009). Cold-activated brown adipose tissue in healthy men.. N Engl J Med.

[14] Virtanen KA, Lidell ME, Orava J, Heglind M, Westergren R (2009). Functional brown adipose tissue in healthy adults.. N Engl J Med.

[15] Rothwell NJ, Stock MJ (1979). A role for brown adipose tissue in diet-induced thermogenesis.. Nature.

[16] Gerencser AA, Neilson A, Choi SW, Edman U, Yadava N (2009). Quantitative microplate-based respirometry with correction for oxygen diffusion.. Analytical chemistry.

[17] Bielohuby M, Menhofer D, Kirchner H, Stoehr BJ, Muller TD (2011). Induction of ketosis in rats fed low-carbohydrate, high-fat diets depends on the relative abundance of dietary fat and protein.. American journal of physiology Endocrinology and metabolism.

[18] Stock MJ (1999). Gluttony and thermogenesis revisited.. Int J Obes Relat Metab Disord.

[19] Feldmann HM, Golozoubova V, Cannon B, Nedergaard J (2009). UCP1 ablation induces obesity and abolishes diet-induced thermogenesis in mice exempt from thermal stress by living at thermoneutrality.. Cell Metab.

[20] Caton SJ, Yinglong B, Burget L, Spangler LJ, Tschop MH (2009). Low-carbohydrate high-fat diets: regulation of energy balance and body weight regain in rats.. Obesity (Silver Spring).

[21] Bielohuby M, Sawitzky M, Stoehr BJ, Stock P, Menhofer D (2011). Lack of dietary carbohydrates induces hepatic growth hormone (GH) resistance in rats.. Endocrinology.

[22] Hondares E, Iglesias R, Giralt A, Gonzalez FJ, Giralt M (2011). Thermogenic activation induces FGF21 expression and release in brown adipose tissue.. The Journal of biological chemistry.

[23] Hondares E, Rosell M, Gonzalez FJ, Giralt M, Iglesias R (2010). Hepatic FGF21 expression is induced at birth via PPARalpha in response to milk intake and contributes to thermogenic activation of neonatal brown fat.. Cell Metab.

[24] Commins SP, Watson PM, Levin N, Beiler RJ, Gettys TW (2000). Central leptin regulates the UCP1 and ob genes in brown and white adipose tissue via different beta-adrenoceptor subtypes.. The Journal of biological chemistry.

[25] Adam-Perrot A, Clifton P, Brouns F (2006). Low-carbohydrate diets: nutritional and physiological aspects.. Obesity reviews: an official journal of the International Association for the Study of Obesity.

[26] Rodriguez AM, Roca P, Palou A (2002). Synergic effect of overweight and cold on uncoupling proteins expression, a role of alpha(2)/beta(3) adrenergic receptor balance?. Pflugers Archiv: European journal of physiology.

[27] Romestaing C, Piquet MA, Bedu E, Rouleau V, Dautresme M (2007). Long term highly saturated fat diet does not induce NASH in Wistar rats.. Nutrition & metabolism.

[28] Portillo MP, Serra F, Simon E, del Barrio AS, Palou A (1998). Energy restriction with high-fat diet enriched with coconut oil gives higher UCP1 and lower white fat in rats.. International journal of obesity and related metabolic disorders: journal of the International Association for the Study of Obesity.

[29] Nomura S, Ichinose T, Jinde M, Kawashima Y, Tachiyashiki K (2008). Tea catechins enhance the mRNA expression of uncoupling protein 1 in rat brown adipose tissue.. The Journal of nutritional biochemistry.

[30] Petzke KJ, Riese C, Klaus S (2007). Short-term, increasing dietary protein and fat moderately affect energy expenditure, substrate oxidation and uncoupling protein gene expression in rats.. The Journal of nutritional biochemistry.

[31] Takahashi Y, Ide T (2000). Dietary n-3 fatty acids affect mRNA level of brown adipose tissue uncoupling protein 1, and white adipose tissue leptin and glucose transporter 4 in the rat.. The British journal of nutrition.

[32] Becerril S, Rodriguez A, Catalan V, Sainz N, Ramirez B (2010). Deletion of inducible nitric-oxide synthase in leptin-deficient mice improves brown adipose tissue function.. PLoS One.

[33] Ghorbani M, Himms-Hagen J (1997). Appearance of brown adipocytes in white adipose tissue during CL 316,243-induced reversal of obesity and diabetes in Zucker fa/fa rats.. International journal of obesity and related metabolic disorders: journal of the International Association for the Study of Obesity.

[34] Liu X, Rossmeisl M, McClaine J, Riachi M, Harper ME (2003). Paradoxical resistance to diet-induced obesity in UCP1-deficient mice.. J Clin Invest.

[35] Blessing W, Mohammed M, Ootsuka Y (2012). Heating and eating: Brown adipose tissue thermogenesis precedes food ingestion as part of the ultradian basic rest–activity cycle in rats.. Physiology & Behavior.

[36] Gomez-Sierra JM, Canela EI, Esteve M, Rafecas I, Closa D (1993). Analysis of ultradian heat production and aortic core temperature rhythms in the rat.. Archives internationales de physiologie, de biochimie et de biophysique.

[37] Sadurskis A, Dicker A, Cannon B, Nedergaard J (1995). Polyunsaturated fatty acids recruit brown adipose tissue: increased UCP content and NST capacity.. The American journal of physiology.

[38] Fromme T, Klingenspor M (2011). Uncoupling protein 1 expression and high-fat diets.. American journal of physiology Regulatory, integrative and comparative physiology.

[39] Brito MN, Brito NA, Migliorini RH (1992). Thermogenic capacity of brown adipose tissue is reduced in rats fed a high protein, carbohydrate-free diet.. The Journal of nutrition.

[40] Moura MA, Kawashita NH, Brito SM, Brito MN, Kettelhut IC (2001). Effect of cold acclimation on brown adipose tissue fatty acid synthesis in rats adapted to a high-protein, carbohydrate-free diet.. Metabolism: clinical and experimental.

[41] Rothwell NJ, Stock MJ (1987). Influence of carbohydrate and fat intake on diet-induced thermogenesis and brown fat activity in rats fed low protein diets.. The Journal of nutrition.

[42] Bachman ES, Dhillon H, Zhang CY, Cinti S, Bianco AC (2002). betaAR signaling required for diet-induced thermogenesis and obesity resistance.. Science.

[43] Whittle AJ, Carobbio S, Martins L, Slawik M, Hondares E (2012). BMP8B Increases Brown Adipose Tissue Thermogenesis through Both Central and Peripheral Actions.. Cell.

[44] Rothwell NJ, Stock MJ, Tyzbir RS (1983). Mechanisms of thermogenesis induced by low protein diets.. Metabolism: clinical and experimental.

[45] Schwartz JH, Young JB, Landsberg L (1983). Effect of dietary fat on sympathetic nervous system activity in the rat.. The Journal of clinical investigation.

[46] Puigserver P, Spiegelman BM (2003). Peroxisome proliferator-activated receptor-gamma coactivator 1 alpha (PGC-1 alpha): transcriptional coactivator and metabolic regulator.. Endocrine reviews.

[47] Coulter AA, Bearden CM, Liu X, Koza RA, Kozak LP (2003). Dietary fat interacts with QTLs controlling induction of Pgc-1 alpha and Ucp1 during conversion of white to brown fat.. Physiological genomics.

[48] Seale P, Kajimura S, Yang W, Chin S, Rohas LM (2007). Transcriptional control of brown fat determination by PRDM16.. Cell Metab.

[49] Ohlson KB, Mohell N, Cannon B, Lindahl SG, Nedergaard J (1994). Thermogenesis in brown adipocytes is inhibited by volatile anesthetic agents. A factor contributing to hypothermia in infants?. Anesthesiology.

[50] Kienbaum P, Heuter T, Michel MC, Peters J (2000). Racemic ketamine decreases muscle sympathetic activity but maintains the neural response to hypotensive challenges in humans.. Anesthesiology.

[51] Ohlson KB, Shabalina IG, Lennstrom K, Backlund EC, Mohell N (2004). Inhibitory effects of halothane on the thermogenic pathway in brown adipocytes: localization to adenylyl cyclase and mitochondrial fatty acid oxidation.. Biochemical pharmacology.

[52] Ma SW, Foster DO (1989). Brown adipose tissue, liver, and diet-induced thermogenesis in cafeteria diet-fed rats.. Canadian journal of physiology and pharmacology.

[53] Ma SW, Foster DO, Nadeau BE, Triandafillou J (1988). Absence of increased oxygen consumption in brown adipose tissue of rats exhibiting “cafeteria” diet-induced thermogenesis.. Can J Physiol Pharmacol.

