# Supplementary material for: Isoenergetic Feeding of Low Carbohydrate-High Fat Diets Does Not Increase Brown Adipose Tissue Thermogenic Capacity in Rats
Source: PLoS One. 2012 Jun 13;7(6):e38997. doi: 10.1371/journal.pone.0038997 (PMC3374780; doi:10.1371/journal.pone.0038997)
Supplement: Table S1 — Average amount of lipid in tissue section (percentage of high power field). (DOCX) [file pone.0038997.s001.docx]

**Table S1:**

Average amount of lipid in tissue section (percentage of high power field)

|  | Control | LC-HF-LP | LC-HF-NP | High fat |
| --- | --- | --- | --- | --- |
| Average±SEM % | 62.9±2.2 | 69.8±0.9 | 69.9±0.9 | 67.4±1.9 |
| p vs. control (Dunnett test) |  | 0.025 | 0.032 | 0.148 |
| Global ANOVA p=0.037 | | | | |
