# Supplementary material for: Isoenergetic Feeding of Low Carbohydrate-High Fat Diets Does Not Increase Brown Adipose Tissue Thermogenic Capacity in Rats
Source: PLoS One. 2012 Jun 13;7(6):e38997. doi: 10.1371/journal.pone.0038997 (PMC3374780; doi:10.1371/journal.pone.0038997)
Supplement: Table S2 — Major effects of the experimental diets compared to control. (DOCX) [file pone.0038997.s002.docx]

**Table S2:**

Major effects of the experimental diets compared to control
