# Supplementary material for: Isoenergetic Feeding of Low Carbohydrate-High Fat Diets Does Not Increase Brown Adipose Tissue Thermogenic Capacity in Rats
Source: PLoS One. 2012 Jun 13;7(6):e38997. doi: 10.1371/journal.pone.0038997 (PMC3374780; doi:10.1371/journal.pone.0038997)
Supplement: Methods S1 — Quantification of surface covered by lipids. (DOCX) [file pone.0038997.s003.docx]

**Supplementary methods**

*Quantification of surface covered by lipids*

Images of HE stained interscapular BAT sections were taken at 40x magnification and 10 mega pixels resolution. Quantification of the surface of lipid droplets was quantified using ImageJ, Version 1.44 (NIH, Bethesda). Images were converted to 8-bit gray scale and brightness. Automatic thresholding of the image was performed using Otsu’s histogram based algorithm. White lipid areas were assigned the value 255, rest of the image black assigned the pixel value 0. Measurement of lipid area with ImageJ “measurement” command, output: mean pixel value over all image pixels. Lipid surface was calculated by mean divided by 255 multiplied by 100 to yield the percentage of lipid surface per high power field. N=4 per diet group, 4 images per animal were analyzed and averaged for calculation. Statistical significance was tested by one-way ANOVA and Dunnett test.
